# Implementing an ICT-Based Polypharmacy Management Program in Italy

**Published:** 2017-07-01

**Authors:** M. Arcopinto, M. Cataldi, V. De Luca, V. Orlando, G. Simeone, R. D’Assante, A. Postiglione, A. Guida, U. Trama, M. Illario, N. Ferrara, E. Coscioni, G. Iaccarino, P. Cuccaro, G. D’Onofrio, C. Vigorito, A. Cittadini, E. Menditto

**Affiliations:** 1 Dipartimento Assistenziale ad Attività Integrata di Medicina Interna e Patologia Clinica, Dipartimento di Scienze Mediche Traslazionali, Divisione di Medicina Interna e Riabilitazione Cardiologica, Università di Napoli Federico II, Milano, Italy; 2 Dipartimento di Neuroscienze, Scienze Riproduttive ed Odontostomatologiche, Divisione di Farmacologia, Università di Napoli Federico II, Milano, Italy; 3 U.O.S. Ricerca e Sviluppo, Azienda Ospedaliera Universitarià Federico II, Milano, Italy; 4 Facoltà di Farmacia, CIRFF/Centro di Farmacoeconomia, Università di Napoli Federico II, Milano, Italy; 5 Dipartimento di Cardiochirurgia, IRCCS Policlinico San Donato, Milano, Italy; 6 Divisione di Geriatria, Dipartimento di Scienze Mediche Traslazionali, Divisione di Medicina Interna e Riabilitazione Cardiologica, Università di Napoli Federico II, Salerno, Italy; 7 Azienda Ospedaliera Universitaria OO.RR. San Giovanni di Dio Ruggi d’Aragona, Salerno, Italy; 8 Dipartimento di Medicina, Chirurgia e Odontoiatria, Scuola Medica Salernitana, Università di Salerno, Italy; 9 Direzione Sanitaria, Azienda Ospedaliera Universitaria Federico II, Napoli, Italia

**Keywords:** polypharmacy, elderly patients

## Abstract

Although there is evidence of a growing awareness of the problem, no official policy statements or regulatory guidelines on polypharmacy have been released up to date by Italian Health Authorities. Medication review, application of appropriateness criteria and computerized prescription support systems are all possible approaches in order to improve the quality of prescribing in older persons. More focused training courses on multimorbidity and polytherapy management are encouraged. Furthermore a multidisciplinary approach integrating different health care professionals (physicians, pharmacists, and nurses) may positively impact on reducing the sense of fear related to discontinue or substitute drugs prescribed by others; the fragmentation of therapy among different specialists; reducing costs; and improving adverse drug reaction detection and reporting. Aiming at achieving the individualized pharmacotherapy, a multidisciplinary approach starting with identification of patients and risk for drug-related problems, followed by medication review overtime and use of inappropriateness criteria, supported by computerized systems has been proposed.

## I. INTRODUCTION

Polypharmacy is the use of four or more medications by a patient [1]. Polypharmacy is more commonly observed in patients older than 65, affecting about 40% of not institutionalized older adults. About 20% of adults with general learning disability are also exposed to polypharmacy, which is often associated with a decreased quality of life, and with mobility and cognitive disorders [2–6]. Polypharmacy also increases the risk of adverse drug reactions (ADR) and of drug interactions, and may, therefore, trigger a prescribing cascade and increase the costs of therapy [7].

The global economic and financial crisis is negatively impacting on the Italian healthcare system, which is undergoing a devolution process from the central to regional governments that in about one third of the the lack in Italy of programs on polypharmacy management or drug adherence. Taking into account the aforementioned scenario, the present review aims to provide an evidence-based description of the current status of polypharmacy in older adults in Italy.

## II. POLYPHARMACY: MOVING FROM IMPORTANCE TO URGENCY

The heterogeneity in health policies among different Italian regions is a likely causative factor of the lack in Italy of key policies and procedures on polypharmacy management in older adults. In fact, Italy is characterized by a National Health System, which is differently designed, administered and organized at the regional level. Therefore, local issues on inequalities in health services utilization can be ascribed to the regional context and data extension at the national level are eagerly awaited. In Italy, the delivery of care is mostly hospital centred, with a consequent lack of resources available for territorial care. The latter is delivered mostly by general practitioners. A recent legislation has modified the territorial care in Italy by introducing the concept of primary care, and by establishing the so called Complex Unity for Primary Care (UCCP) as a new tool to enhance the integration among different healthcare professionals (general practitioner, clinical nurses, territorial specialists, obstetricians, socio-sanitary personnel) and to facilitate the delivery of territorial care. Beside the difficulties experienced by the individual regions in applying such model, the law does not include clinical pharmacists among the professionals of the UCCPs. This remains a missed opportunity also considering that the previous law no. 69/2009 acknowledged the relevance of clinical pharmacists by defining the role of community pharmacies as socio-sanitary multipurpose service centres which should help to tackle problems related to chronic clinical conditions and polypharmacy hence playing a complementary role with UCCP. Unfortunately, community pharmacies are not currently providing patients with “pharmaceutical” care, but only with dispensation of drugs. This depends on a number of factors including the slow raise of political interest in promoting the role of pharmacists as caregivers, the absence of financial investments to support this kind of service and the poor organization and communication between stakeholders both at local and national level.

## III. AWARENESS OF THE SCIENTIFIC COMMUNITY ON POLYPHARMACY-RELATED ISSUES IN ITALY

In the last decade, several studies investigated the quality of drug prescriptions and other polypharmacy-related issues in older adults in Italy. The Geriatric working Group of the Italian Medicines Agency (AIFA), the national authority responsible for drug regulations in Italy, conducted a study on the quality of drug prescription in the older (65+ years of age) Italian population providing a representative snapshot of pharmacological treatment in this age group [12]. This study focused on 13 quality indicators to measure polypharmacy, adherence to the treatment of chronic diseases, the occurrence of prescribing cascades, undertreatment, and drug-drug interactions. Polypharmacy was common in this age group and about 1.3 out of the 11.5 millions of the tested individuals was taking 10 or more drugs (around 11% of the entire cohort). The prevalence of low adherence and undertreatment was also quite high and it proportionally increased with advancing age, peaking in individuals aged 85 years and older.

The “Gruppo Italiano di Farmacovigilanza nell’Anziano” (GIFA) study was a collaborative pharmacosurveillance study on hospitalized patients that was sponsored by the Italian National Research Council and the Italian Society of Gerontology and Geriatrics with the aim of studying quality of care and problems related to pharmacological therapy in older adults [13]. From 1988 to 1998 the GIFA study evaluated more than 30,000 hospital admissions of older patients, and found that about 3.4% of them were ADR-related.

The “REgistro POliterapie Societa’ Italiana di Medicina Interna” (REPOSI) registry is a collaborative research project involving the Italian Society of Internal Medicine, the IRCSS Ca Grande Maggiore Policlinico Hospital Foundation of Italy and the IRCSS Istituto di Ricerche Farmacologiche Mario Negri of Milan [14]. The REPOSI registry is a network of internal Medicine and geriatric wards that was established in order to evaluate comorbid geriatric patients treated with polypharmacy. The specific aims of the REPOSI registry were measuring the prevalence of comorbidity and treatments in hospitalized older adults, correlating clinical characteristics of patients with type and number of diseases and treatments, and evaluating primary clinical outcomes at hospital discharge and adverse events during hospitalizations. In addition, data from REPOSI registry were used to evaluate the association of specific diseases with polypharmacy [15]. Data showed that quality of prescriptions in patients discharged from hospitals was poor, and that often elderly patients with comorbidity did not receive appropriate treatment for chronic diseases.

The criteria to Assess Appropriate Medication Use Among Elderly Complex Patients (CRIME) research project was funded by the Italian Ministry of Labour, Health and Social Policy with the aim of assessing the quality of prescription in hospitalized older adults and of getting helpful information to improve drug prescription in patients with high comorbidity and complex diseases [16]. This observational study performed in the geriatric and internal medicine acute care departments of 7 Italian hospitals collected data from 1123 patient, including their socio-demographic characteristics, several indicators of functional capacity and cognitive status, the clinical diagnoses at admission, and both the drug therapy given in-hospital and prescribed at discharge. The CRIME study showed that both polypharmacy and inappropriate prescribing were extremely common among hospitalized older adults. According to the study’s criteria, more than 50% of drug prescriptions were inappropriate and the prevalence of polypharmacy (defined as concomitant use of ≥8 drug) was about 50%. These conditions negatively impact on health outcomes, such as the occurrence of ADRs, increased length of in-hospital stay, worsening functional status and mortality.

Italy accounts for the lowest number of nursing home (NH) beds for the elderly, although there are marked differences in the availability of NH beds throughout the regions. Few data describing the individual and organizational characteristics of residents in long-term care facilities are available. Given the absence of a validated methodology for routine assessment of long-term care facilities in all Italian regions, this crucial information is needed for implementing regional or national policies.

Recently, Comprehensive Geriatric Assessment was validated and implemented, and the InterRAI tool for the assessment of Long-Term Care Facilities (InterRAI LTCF) became essential for assessing characteristics of NH residents, including drug use, in clinical studies. The Services and Health for Elderly in Long-Term Care (SHELTER) study was a project funded by the 7th Framework Program of the European Union that translated, validated and implemented the interRAI LTCF across different health systems in European countries as a tool to assess and gather uniform information about NH residents [17]. The SHELTER study was conducted from 2009 to 2011 in 7 EU countries (Czech Republic, England, Finland, France, Germany, Italy and the Netherlands) and one non-EU country (Israel). Additionally, the “Un Link Informatico Sui Servizi Sanitari Esistenti per L’anziano” (ULISSE) study implemented the interRAI LTCF in 31 NHs in Italy [18].]

Although these studies were not specifically designed to assess drug use, SHELTER showed that polypharmacy was extremely common in NH residents, and that it was associated with negative outcomes. This held especially true in residents with limited life expectancy. Notably, in these patients, clinical benefits derived from the use of multiple drugs were negligible and did not mitigate the risk of iatrogenic illness. The negative effects of polypharmacy were also confirmed in the ULISSE study, which showed that the concomitant use of multiple drugs was associated with an increased risk of hospitalization.

Although the available data suggests that polypharmacy could be efficiently tackled, public health strategies on this specific issue are still missing.

## IV. CHANGE MANAGEMENT STRATEGIES

Therapeutic Review and Medication Reconciliation are two mandatory steps both in drug prescribing and in drug de-prescribing (tapering or stopping drugs) processes. In fact, the same good practices and principles should be applied when a drug therapy is initiated and when it is time that it is discontinued [19].

Therapeutic review is a systematic process in which physicians collect information on all conventional and non-conventional drugs taken by a specific patient, and compare them with those recommended by guidelines. At the time of therapeutic review potential drug-related problems in patient therapeutic regimen are also identified. The final aim of therapeutic review is to verify whether patient’s current medications are appropriate and have a favourable benefit-to-risk ratio. Usually, medication review is a multidisciplinary process involving not only the prescribing physician, but also the clinical pharmacist and other health care professionals such as clinical nurses. Thanks to the contribution of these diverse professional benefits and risks of the drug treatment in place are evaluated under multiple perspectives and possible solutions for potential drug-related problems are identified and their feasibility and applicability is critically examined [20]. Additional benefits of therapeutic review include an increase in patient safety due to reduced discrepancies, improved prescription appropriateness and reduced ADRs.

Medication reconciliation is the process of avoiding inadvertent inconsistencies within a patient’s drug regimen, which can occur during transitions in different setting of care. These reviews are conducted to ensure that the patient is still on the appropriate drug therapy regimen after experiencing care transitions [21]. The role of medication reconciliation as a routinal and standardized step, especially in the transitional care process, is supported by health care agencies at both European level and the national level. For example, both the Joint Commission and the Italian Ministry of Health have acknowledged medication reconciliation as an important tool to prevent medical errors and drug-related problems [22]. Medication reconciliation is essential for the continuity of patient’s treatment across different hospitals, hospital wards and specialists, and when the patient returns to primary care after being discharged from the hospital. This does not mean, however, that the drug regimen has to be indefinitely kept unchanged. Indeed, medication reviews mainly checks for inadvertent or erronoeus changes but drug prescription should be viewed as a dynamic process in which drug benefits or harms are continously checked, managed and assessed in a multidisciplinar and integrated longitudinal process. Moreover, the approach should incorporate implementation of inappropriate drug criteria and computer-based prescribing systems.

Still, specific tools to assess quality of prescribing and to avoid inappropriate drug prescriptions have not been yet implemented. During the last few decades, much effort has been produced to improve the quality of drug prescription in older adults, and several criteria have been developed [23–26]. However, the lack of data integration and interoperability of ICT solutions in healthcare is a national issue in Italy that complicates their widespread application. Indeed, in 2016, Italy is still lacking a nationwide e-health record. In addition, pilot studies do not translate in regulatory actions of the National Health System.

Clinical practice guidelines (CPGs) are an additional tool that could help change management strategies. CGPs are condition-specific recommandations that are developed to support physicians in the decision-making processes. The movement towards evidence-based medicine quickly widespread over the past few years, stimulated by clinicians, politicians and decision-makers concerned about quality, consistency and costs. The guidelines content, based on a systematic review of clinical studies, have been shown to be effective in supporting improvements in quality and consistency in healthcare. In Italy, the National System for Guidelines [Sistema Nazionale Linee Guida (SNLG)] give clinical recommendations based on the most updated researches [27]. Unfortunately, to date, no specific tools, guidelines or policies for polipharmacy management and monitoring in Italy are available. The importance of such tools is of paramount importance in the light of the multiple drug therapies also including hormones in some patient populations, such as subjects with chronic heart failure [28, 29].

## V. AN ICT-BASED MODEL TO TACKLE POLYPHARMACY RELATED ISSUES

Every single patient needs a comprehensive assessment in order to give him a personalized therapy that balances benefits and harms and takes full account of his priorities and preferences. During this patient-specific assessment medical doctors have to evaluate the numerous factors that can influence the efficacy of the drugs that they are going to prescribe and that are not covered in disease-specific guidelines. In older patients, these factors could include, for instance, cognitive and functional impairment, and the lack of social support. Because there are too many potential drug-drug interactions across all the possible combinations of the drugs used for chronic disease to be memorized by the average medical doctor or to be included in clinical practice guidelines, efficient computerised prescription support tools are needed to alert the physicians on problems potentially caused by their prescriptions, helping them to improve drug prescribing and to reduce the risk of harmful drug interactions.

Computerized Entry Order and Computerized Prescription Support Systems are interactive software systems created to assist physicians in providing the correct prescriptions, and ultimately avoiding drug-related problems. Computerized prescription support systems might change health care provider behaviour, improving physicians performance, and reducing drug-drug interactions and the number of inappropriate prescriptions [30]. However, these methods are rarely used in combination with multidimensional approach tools to assess the complexity of older patients such as the Comprehensive Geriatric Assessment (CGA) tool although a number of studies showed that CGA is the gold-standard method to manage and improve the quality of prescriptions [31]. Preliminary data demonstrated that CGA, when combined with the computerized approaches, improves drug use and has positive effects on health outcomes, including a reduction in ADRs. A “global approach” also addressing the medical complexity of older adults seems, therefore, to be mandatory.

The final goal to achieve should be to deliver a tailored pharmacotherapy that takes into the due account some critical issues, such as patients’ characteristics, life expectancy, preferences and priorities. Time to benefit and the relative effectiveness of treatment in specific clinical conditions should also be considered. Facilitators and barriers for the implementation of such a polypharmacy and adherence review program are summarized in Tables 1 and 2.

**Figure 1:**
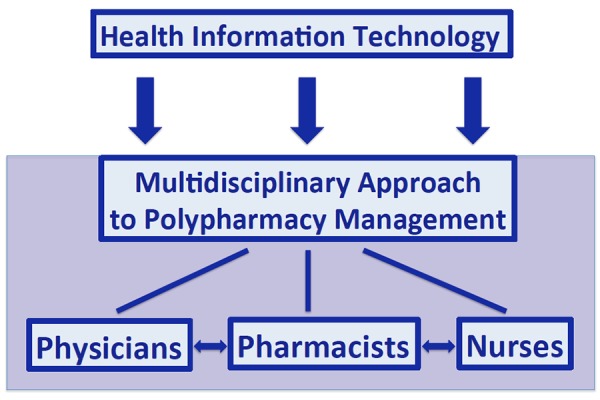
Proposed algorithm for polypharmacy management To deliver the best-tailored pharmacotherapy for each single patient a multicomponent approach should be adopted. It starts with the identification of patients at risk for drug-related problems that are, then, followed overtime with periodic medication reviews also using drug inappropriateness criteria. This patient care strategy is embedded in the comprehensive geriatric assessment and is supported by computerized systems.

We propose that to achieve the aforementioned goal of a tailored pharmacotherapy a *multifaceted intervention* should be implemented that starts from the idetification of patients at risk of drug-related problems (Figure 1). Risk scores for ADRs are currently available in the literature and are easily applicable in everyday clinical practice. In addition, the medical and/or pharmaceutical personnel in charge of the patient should perform medication review and medication reconciliation, at regular intervals or at any time when she/he changes setting.

Finally, this multifaceted intervention has to be contextualized in the global evaluation of older adults performed with CGA. Frailty, cognitive functioning, symptoms of depression and anxiety, functional status, motor abilities, swallowing problems, socio-economic status, adherence to treatments are all evaluated by the CGA, which, in the end, helps the prescribing physician to choose the best possible and most appropriate pharmacotherapy for a specific patient at that moment. Moreover, CGA may help to improve communication, coordination and transparency among clinicians, patients, caregivers and other providers of care, and it represents a useful instrument for personalization of care.

## VI. CONCLUSION

The present review highlighted the absence in Italy of key policies and procedures for polypharmacy management in older adults. We propose that a multicomponent ICT-based approach could help addressing these issues and should be tested in pragmatic, community-dwelling randomized clinical trials. The effectiveness of this intervention should be evaluated not only with respect to the reduction of inappropriate polypharmacy and related-costs, to the improvement in the quality of prescribing or a to a decrease in the burden of drug-related problems, but also with respect to patient-related outcomes and priorities, such as quality of life, hospitalization, emergency room admissions and disability. This strategy could also provide validated evidence regarding better treatment strategies for older adults with multiple diseases.

## Figures and Tables

**Table 1:** Facilitators of the implementation of polypharmacy and adherence review programs

▪ Pilot studies specifically focused on polypharmacy and adherence programme should be designed and implemented to generate evidences on health outcomes in older adults; ▪ University training courses specifically focused on polypharmacy and adherence programme should be implemented and delivered to medicine and pharmacy students, and to nurses; ▪ Healthcare Professional Councils should support the initiatives related to polypharmacy and adherence review programs; ▪ Pharma industries should be involved to the initiatives related to polypharmacy and adherence review programs; ▪ GPs should be consortiated in order to reduce to workload and co-adjuvanted by other health care professionals such as pharmacists and nurses; ▪ In line with other Countries, *clinical pharmacist* (working on medical wards) should be adopted as health care professional in Italy; ▪ Dedicated in-hospital ambulatories on polypharmacy and adherence review; ▪ Community pharmacies should be integrated to GPs for polypharmacy and adherence review programs; ▪ Health information technology infrastructure may help to support implementation and monitoring of a programme.

**Table 2:** Barriers to the implementation of polypharmacy and adherence review programs

▪ Lack of clear policies; ▪ Resistance to change (cultural barriers); ▪ Spending review (poor resources); ▪ Organizational boundaries as silos; ▪ Healthcare system devolution
